# Phenotypic Characterization of Recently Identified PSEN1 Genetic Variants in ADAD in Colombia

**DOI:** 10.1002/alz70861_108984

**Published:** 2025-12-23

**Authors:** Alejandro Guerrero, Elkin Garcia‐Cifuentes, Jan Karlo Rodas‐Marin, Ana Y Baena, Laura Serna, Yudy M Leon‐Varela, Sindy Duque, Juan F Martínez, Yamile Bocanegra, Claudia Muñoz, Laura Ramirez Aguilar, Claudia Ramos, Victoria Zubiri, Jorge J. Llibre‐Guerra, David Aguillon

**Affiliations:** ^1^ Grupo de Neurociencias de Antioquia, Facultad de Medicina, Universidad de Antioquia, Medellín Colombia; ^2^ Department of Neurology, Washington University School of Medicine, St. Louis, MO USA

## Abstract

**Background:**

Thirteen PSEN1 genetic variants associated with Autosomal Dominant Alzheimer’s Disease (ADAD) have been identified in Colombia, providing valuable insights into the preclinical stages of the disease. This study characterizes the clinical and neuropsychological phenotypes of carriers and non‐carriers from families with nine pathogenic *PSEN1* variants: Glu280Ala, Gln223Lys, His163Arg, Ile162Ser, Ile416Thr, Leu173Phe, Pro264Leu, Pro284Leu, and Thr119Ile.

**Method:**

We analyzed baseline clinical and neuropsychological characteristics of participants enrolled in the Dominantly Inherited Alzheimer Network Observational Study (DIAN‐OBS), including individuals at both preclinical and symptomatic stages. Descriptive statistics were used to summarize sociodemographic, clinical, and cognitive features, depending on data distribution, and we used Kruskal‐Wallis to explore differences between groups. Group comparisons were based on PSEN1 variant and clinical diagnosis, and the age at onset (AAO) of the affected parent was used to determine each subject's estimated years to symptom onset (EYO).

**Results:**

The sample included 61 participants (55.7% female), aged 18–58, with a median education of 12 years (IQR: 10–14). Of these, 77% were cognitively unimpaired (MMSE: 30, IQR: 29–30; CDR‐SB: 0), 15% had mild cognitive impairment (MMSE: 25.1, SD: 3.6; CDR‐SB: 1.9, SD: 1.1), and 8% had dementia (MMSE: 21, SD: 6.5; CDR‐SB: 3.9, SD: 3.1). All symptomatic individuals exhibited primarily amnestic symptoms, and one carrier of the PSEN1‐Pro284Leu variant presented motor signs. As expected—given that most participants were in preclinical stages—no statistically significant differences in clinical or cognitive features were observed across variants. Twenty‐eight participants were in the primary prevention range (EYO: ‐11 to ‐35 years) and thirty‐three in the secondary prevention range (EYO: ‐10 to +13 years). Parental age at onset differed by variant location: intramembranous (50 years, IQR: 46–53), cytoplasmic (40.5 years, SD: 4.6), and extracellular (45 years, IQR: 45–48).

**Conclusion:**

These findings are consistent with existing literature and underscore the value of characterizing preclinical ADAD cohorts, supported by biomarkers, to guide early diagnostic and therapeutic strategies, especially in the context of clinical trials. We expect to have complete fluid and neuroimaging biomarker and follow‐up data for the presentation of these results.

